# Traumatic colostomy evisceration in an AIDS patient with anal cancer

**DOI:** 10.1093/jscr/rjaa544

**Published:** 2021-12-24

**Authors:** Uju Momah, Josh Barnaby, Constantine Poulos, Robert Lewis

**Affiliations:** The University of Connecticut School of Medicine, Farmington, CT, USA; Department of General Surgery, Saint Francis Hospital and Medical Center, Hartford, CT, USA; Department of General Surgery, Saint Francis Hospital and Medical Center, Hartford, CT, USA; Department of General Surgery, Saint Francis Hospital and Medical Center, Hartford, CT, USA

## Abstract

Intestinal evisceration is a rare event and few cases of colostomy rupture have been documented in the medical literature. Complications of colostomy surgery vary in incidence, with most episodes occurring in the immediate postoperative timeframe, including necrosis, hemorrhage, cellulitis and dehiscence. Here, we document the case of a 35-year-old male patient with a history of immunodeficiency, multiple comorbidities and squamous cell carcinoma of the anus who experienced a unique instance of colostomy evisceration weeks after initial surgery. The patient originally underwent surgery for a sigmoid colostomy for the alleviation of irritation secondary to anal disease. Weeks later, after a traumatic fall injury, he experienced colostomy evisceration. This case will review the factors leading up to this event that put the patient at risk for poor wound healing and ultimately colostomy rupture.

## INTRODUCTION

Colostomy creation is a common procedure performed for the management of acquired conditions of the bowel or anus. It is associated with several complications, scarcely of which can include stoma evisceration. The etiology, development and management of stoma evisceration have been poorly documented in the literature. This uncommon complication was found in our patient after a mechanical fall. The presentation was complicated by several comorbidities, including Acquired Immunodeficiency Disease Syndrome (AIDS), Kaposi sarcoma, diabetes mellitus, malnutrition, as well as several other conditions. Although traumatic stoma evisceration is rare, it is an important complication of colostomy surgery that requires subsequent surgical repair. Here, we discuss the patient’s hospital course, risk factors predisposing to stoma evisceration and impaired wound healing, as well as options for clinical management. Our aim is to call attention to a rare complication of in patients with intestinal ostomy and highlight a safe approach to management.

## CASE REPORT

The patient was a 35-year-old male with a history of AIDS secondary to intravenous drug use, Kaposi sarcoma, diabetes mellitus, seizure disorder and prior history of infection from several AIDS-defining organisms, including *Mycobacterium avium* complex and *Mycobacterium avium* intracellulare colitis.

**Figure 1:**
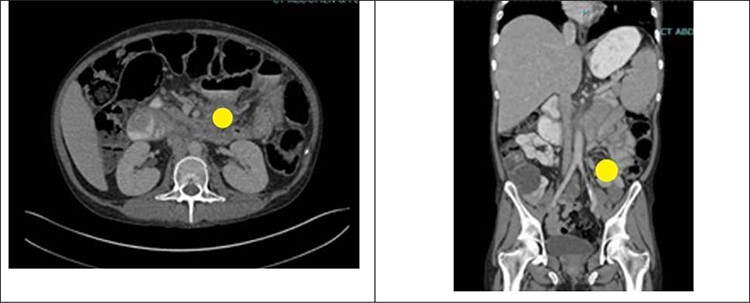
CT abdomen and pelvis with IV contrast showing an ill-defined mass at the root of the mesentery measuring at least 5.2 × 5.1 cm (yellow circle). This was hypothesized to represent conglomerate lymphadenopathy, a potential neoplasm, such as mesothelioma or lymphoma, or metastatic disease. There was also a large volume of abdominal ascites and mildly dilated loops of small bowel within the left abdomen which likely represented a partial obstruction or ileus.

Prior to the index presentation, the patient presented to the emergency department with generalized weakness and blood per rectum for 2 weeks. He had no changes in diet, but endorsed intermittent abdominal discomfort, as well as increasing anal pain and drainage. The only abnormality on digital rectal examination was minor peri-rectal excoriation. Computed tomography CT imaging (Fig. 1) shows significant ascites, a non-obstructing mass at the base of the mesentery rectum, mesenteric adenopathy and dilated loops of small bowel. There were no signs of obstruction or abdominal distension. Evaluation under anesthesia demonstrated a large, firm indurated necrotic mass above the dentate line forming a malignant fistula to the perianal skin. There was also thickening of the perianal skin circumferentially that was likely secondary to chronic irritation from fistulous drainage or pagetoid spread of a malignancy. Biopsy results revealed poorly differentiated invasive squamous cell carcinoma with extensive necrosis. Left and right lateral perianal skin showed focal severe squamous dysplasia and high-grade squamous dysplasia, respectively. Ultimately, the diagnosis of stage T3N0M0 squamous cell carcinoma of the anus was made.

Given sphincter involvement, the decision was made to proceed with diverting sigmoid colostomy for palliation of expected incontinence and alleviation of perianal irritation. The patient’s postoperative disease course was complicated by pancytopenia, ileus and massive ascites (requiring frequent paracenteses), adrenal insufficiency (requiring hydrocortisone), protein malnutrition and significant weight loss.

**Figure 2:**
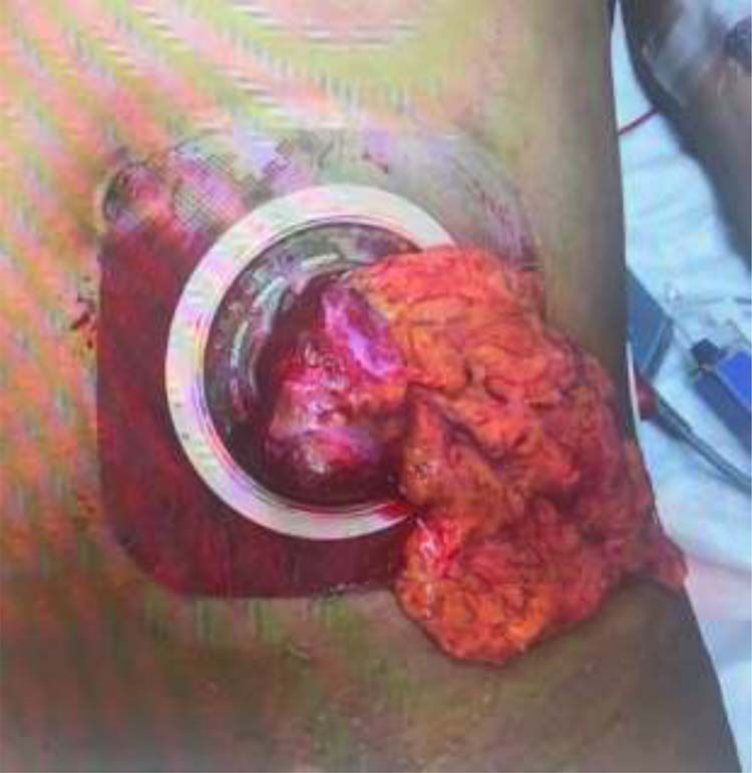
Colostomy site with clear disruption following traumatic fall. Omental fat and a portion of the colon are noted to be eviscerated through the stomal site. The bowel is loose and freely mobile.

**Figure 3:**
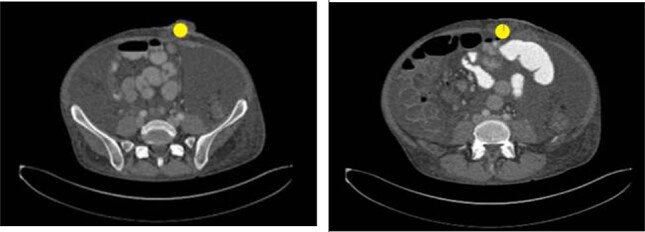
Image taken upon presentation to the emergency department with left lower quadrant ostomy evisceration marked (yellow circle). Oral contrast reaches the mid small bowel. Scattered small bowel loops are mildly prominent measuring up to 3.7 cm in diameter. The colon appears thick-walled particularly along the ascending aspect. There is no pneumatosis. There is no discrete hernia. Findings are consistent with small bowel obstruction.

Seven weeks after colostomy creation, the patient presented as a core trauma activation due to a mechanical fall that resulted in colostomy disruption and evisceration of abdominal contents (Figs 2 and 3). The patient reported a fall down several stairs where he struck his abdomen. On presentation, he was neurologically intact and hemodynamically stable. He was found to have no additional traumatic injuries after workup was completed.

In the emergency room, the patient was frail, cachectic and chronically ill appearing. He denied abdominal pain, nausea, vomiting, shortness of breath, confusion and headache. On exam, there was frank blood in his ostomy bag and visible bleeding from the exposed colon and omentum. There was also visible colonic and omental evisceration, with the ostomy dislodged nearly 75% from the surrounding skin. Additionally, he had a protuberant abdomen and fluid wave, with ascites leaking through the ostomy site. The patient was emergently taken to the operating room for ostomy revision. Under general anesthesia, the eviscerated bowel was amputated, and the ostomy was recreated in the usual fashion without complication. The patient’s immediate postoperative course was uneventful. However, in the coming weeks, he returned to the emergency department with multiple episodes of abdominal discomfort and blood per ostomy. He was followed for several months and ultimately expired due to decline in the status of his concomitant conditions.

## DISCUSSION

The creation of a stoma is a commonly performed surgery utilized for benign and malignant intestinal conditions. Its creation generally yields a 6–59% complication rate [1]. There are several well-known complications associated with the creation of an abdominal stoma, including skin irritation, ischemia, high ostomy outputs, retraction, parastomal herniation, stenosis and prolapse. One rare complication that is infrequently reported is that of stoma evisceration. This complication can occur at any point after the initial stoma surgery; however, of the cases that have been documented, many have been reported within a few days of the initial procedure [2]. Evisceration occurring in the immediate postoperative period is usually attributed to suture disruption along the mucocutaneous surface [2]. Many factors can predispose to evisceration, including the use of corticosteroids, colostomy prolapse, increased intra-abdominal pressure, coughing during the postoperative period, chronic obstructive pulmonary disease, smoking, alcohol abuse and malignant colorectal neoplastic disease [2]. Other important contributing factors include age, obesity and location of the stoma outside the rectus abdominis muscle.

Specifically in our patient, his history was significant for immunocompromise secondary to AIDS, anal cancer and immunosuppressive therapy (active chemotherapy and steroid use secondary to pre-operative adrenal insufficiency), as well as diabetes mellitus, protein malnutrition, ascites, intravenous drug use and smoking.

In a patient with the aforementioned comorbidities, the following complications can occur that impair wound healing:

- Immunocompromise: Chemotherapeutic drugs delay wound healing through suppression of inflammation, rapid cell division and angiogenesis, as well as interruption of cell migration to wound sites. By impeding DNA, RNA and protein synthesis, these agents impair neovascularization and fibroplasia of wounds. Impairment of wound healing most commonly occurs pre-operatively or within 3 weeks after surgery [3]. Similarly, the administration of systemic steroids inhibits wound healing through suppression of fibroblast proliferation, collagen synthesis and hypoxia-inducible factor. Such effects cause wounds to heal with incomplete granulation tissue and reduced wound contraction [3]. Both agents can also predispose to infection, further compromising the wound healing process.
- Malnutrition: Poor protein intake can compromise processes important for wound healing, such as capillary formation, fibroblast proliferation, tissue remodeling and collagen production. In particular, collagen, a major component of connective tissue, requires several amino acids and co-factors including glycine, proline, lysine, glutamine, arginine, copper, ferrous iron and vitamin C, among others. Deficiency of these compounds interferes with collagen synthesis and maturation [3]. In our patient, malnutrition was secondary to both AIDS and cancer.
- Diabetes mellitus: Diabetes, especially if uncontrolled, when combined with the stress of surgery and anesthesia, results in stress hyperglycemia. Elevated glucose levels can promote bacterial proliferation and impair wound healing. Diabetes is also associated with impaired immunity through decreased chemotaxis and phagocytosis, making wounds prone to infection due to compromise of bacterial clearance [4]. Additionally, diabetes interferes with levels of growth factors required for angiogenesis and neovascularization, including vascular endothelial growth factor, angiopoietin-1 and angiopoietin-2, promoting dysgenesis [4]. Diabetics are also prone to altered collagen production, predisposing to weakened wound tensile strength [5].
- Smoking: Smoking promotes tissue ischemia in the bowel by compromising vascular integrity, tissue perfusion and oxygen delivery, which together can prolong wound healing and potentiate stoma detachment. Smoking also disrupts epithelial regeneration, integrity of the extracellular matrix, and angiogenesis. Additionally, nicotine promotes vasoconstriction and when this occurs, in conjunction with catecholamine release stimulated by tobacco, promotes hypoxia [4].

Targets of intervention to improve wound healing secondary to the above risk factors should include the following:

- Immunotherapy: Impaired wound healing is a known complication of chemotherapy, particularly due to its antiangiogenic effects. Therefore, chemotherapeutic agents should be temporarily discontinued preoperatively. Specifically, tyrosine kinase inhibitors should be stopped for 1 week prior to surgery and not restarted until sufficient wound healing has occurred [6]. In general, if chemotherapy is necessary, their inhibitory effect on wound healing can be mitigated by reinitiating administration 10-14 days after wound closure [5]. Additionally, corticosteroids should be avoided in patients due to their known effects on wound healing. In the case of patients with prior systemic corticosteroid exposure, vitamin A (15 000–25 000 IUs daily, limited to 4 weeks) has been shown to improve wound healing by enhancing wound tensile strength [4].
- Malnutrition: Inherently surgery induces a physiologic stress on the body. When surgery is further complicated by the presence of inflammatory disease and malignancy, recovery becomes more difficult due to the elevated turnover of body proteins and anabolic processes. Therefore, nutritional status should be assessed preoperatively with serum pre-albumin, albumin and transferrin levels. These markers, respectively, predict a negative catabolic state, reflect recent systemic inflammation and improve as malnutrition resolves [7]. Optimizing nutritional status in the perioperative setting is important because it can mitigate the physiologic effects of the surgical stress response, promote optimal recovery and support wound healing. In severely malnourished cancer patients, such as our patient, 7–10 days of preoperative parenteral nutritional support is recommended prior to undergoing major visceral surgery [8].
- Diabetes mellitus: The optimization of perioperative glucose control, as well as long-term glucose management, is imperative to support wound healing. Achieving normoglycemia with insulin therapy can promote fibroblast proliferation, improve collagen synthesis and increase wound tensile strength. It is recommended that oral hypoglycemics be discontinued the morning of surgery and metformin should be withheld 48 hours prior to surgery. After surgery, serum glucose levels should be checked and kept under 110 mg/dL [6].
- Smoking: The discontinuation of smoking is associated with a reduced likelihood of wound infection and healing complications. Cessation for at least 4 weeks prior to surgical intervention is important. Continued cessation in the postoperative period is also crucial to mitigate the ischemic and vasoconstrictive effects of tobacco use and support the process of wound healing. Additionally, the use of nicotine-containing products should be avoided during these periods as well.

Although traumatic ostomy evisceration is rare, it was not entirely unanticipated in a patient like ours given their comorbidities that predisposed to poor wound healing and increased risk for wound dehiscence. In the case of our patient, the best measures to avoid such a complication were to mitigate modifiable (smoking, nutrition, chemotherapy, steroids and diabetes) and non-modifiable (malignancy) risk factors, as previously outlined. Efforts were made to medically optimize the patient, including continuing his anti-retroviral therapy and maintaining compliance with anti-seizure medications. However, despite attempts at optimization, stoma evisceration still occurred, requiring operative intervention. Without treatment, patients are at high risk for morbidity. Current practices recommend the creation of stomas within the rectus muscle to reduce the risk of herniation and promote maintenance of the intervention. To prevent complications, an anastomosis should be created using a highly vascularized, tension-free region between the skin and the intestinal end. The orifice created in the skin and fascia should be large enough to encapsulate the bowel and allow for continued, adequate perfusion to maintain intestinal function. In the case of our patient, the surgical approach taken was that of a normal ostomy creation with 3-0 Vicryl sutures.

At large, this case highlights one of the rare complications of colostomy creation and presents ways to minimize its occurrence. Currently, there are only a few cases of stomal evisceration detailed in the literature. Furthermore, within the available research and clinical cases that have been outlined, there is no absolute consensus for how to approach this challenging problem.

## CONCLUSION

In summary, we report a case of stomal evisceration in a young male with a significant past medical history predisposing him to poor wound healing and surgical complications. Although rare, stomal evisceration can occur in patients with underlying comorbidities. Of key importance is the effort that should be made to maximize factors that promote reparative processes of intrinsic wound healing.

## References

[1] Lolis ED . Parastomal evisceration as an extremely rare complication of a common procedure. Ann R Coll Surg Engl 2015;97:e103–4. 10.1308/rcsann.2015.0017 PMC5210138 26274758

[2] Kulkarni AA . Parastomal evisceration: a report of two cases and review of literature. Cureus 2019;11:e5750. 10.7759/cureus.5750 PMC6825462 31723511

[3] Guo S, DiPietro LA. Factors affecting wound healing. J Dent Res 2010;89:219–29. 10.1177/0022034509359125 PMC2903966 20139336

[4] Wernick B. “Impaired Wound Healing.” *StatPearls*. U.S. National Library of Medicine, 7 September 2020. http://www.ncbi.nlm.nih.gov/books/NBK482254/.

[5] Myers WT . Optimizing the patient for surgical treatment of the wound. Clin Plast Surg 2007;34:607–20. 17967617 10.1016/j.cps.2007.07.002

[6] Armstrong, DD, AJ Meyr. Basic Principles of Wound Healing. *UpToDate*. http://www.uptodate.com/contents/basic-principles-of-wound-healing.

[7] Manzullo, EF . Preoperative Evaluation and Management of Patients with Cancer. *UpToDate*. http://www.uptodate.com/contents/preoperative-evaluation-and-management-of-patients-with-cancer.

[8] Husain, SG, TE Cataldo. Late Stomal Complications. Clin Colon Rectal Surg 2008;21:31–40. 10.1055/s-2008-1055319 PMC2780194 20011394

